# Misaligned or misheard? Physical activity and healthy eating messaging to ethnic minority communities during the COVID-19 pandemic: A qualitative study and scoping review

**DOI:** 10.1371/journal.pgph.0003345

**Published:** 2024-10-03

**Authors:** Olatundun Gafari, Sandra Agyapong-Badu, Nisreen A. Alwan, Mark A. Tully, Suzanne McDonough, Maria Stokes, Mary Barker

**Affiliations:** 1 School of Health Sciences, University of Southampton, Southampton, United Kingdom; 2 NIHR Southampton Biomedical Research Centre, University Hospital Southampton NHS Foundation Trust, Southampton, United Kingdom; 3 School of Sport, Exercise and Rehabilitation Sciences, University of Birmingham, Birmingham, United Kingdom; 4 School of Primary Care and Population Sciences, University of Southampton, Southampton, United Kingdom; 5 School of Medicine, Ulster University, Londonderry, Northern Ireland; 6 School of Physiotherapy, Royal College of Surgeons Ireland, Dublin, Ireland; 7 MRC Lifecourse Epidemiology Research Centre, University of Southampton, Southampton, United Kingdom; The Ohio State University, UNITED STATES OF AMERICA

## Abstract

This mixed-methods study identified physical activity (PA) and healthy eating messages produced during the COVID-19 pandemic and explored how they were received by UK ethnic minority communities. A scoping review of research and grey literature identified categories of PA and healthy eating messaging targeted at ethnic minorities. Individual and group interviews were conducted, audio-recorded, transcribed and analysed using inductive thematic analysis. There was active community engagement in all study phases to ensure relevance and co-production of findings. Interviews were held with 41 study participants aged 18–86 years (20 men) residing in England and Wales using digital conferencing and in person. The scoping review identified 24 records containing messages grouped into three categories: 1) PA messages; 2) healthy eating messages; 3) risk messages. Five themes described participants’ views of these messages: 1) lack of awareness of messaging; 2) responses to PA messaging; 3) responses to healthy eating messaging; 4) perceptions of risk messaging and 5) perceptions of conflict in messages. The review revealed that physical activity and healthy eating messaging specifically targeting ethnic minority communities is limited. This limited messaging was almost entirely missed by these communities. When received, the messaging was not interpreted as intended, perceived to be conflicting and risk messaging was perceived as blaming. More work with ethnic minority communities needs to be done to co-produce meaningful and appropriate PA and healthy eating messaging in a timely manner.

## Introduction

Malnutrition and physical inactivity are two of the most important contributors to the global burden of disease [1]. Regularly engaging in physical activity (PA) and eating a healthy diet improves physical and mental wellbeing [2], prevents chronic conditions [3] and reduces infectious disease risk through improved immune function [4].

There are long-standing health inequalities in the UK between the white majority and ethnic minorities communities, [5], echoing wider social and economic inequalities between these communities [6]. Compared to their White British counterparts, UK EMCs are more likely to be unemployed [7], live in more deprived areas [8] and in overcrowded [9] and low-income households [10], and have less access to health and social care [11]. People from Black and Asian communities are also less likely to be physically active [12] or eat five portions of fruits and vegetables daily [13]. This has long-term health consequences and contributes to their risk of diabetes mellitus [14] and cardiovascular diseases [15], two leading causes of death in the UK; risks which are higher among these communities [16]. Many chronic health conditions of public health significance are modifiable through improved nutrition and PA [17].

The COVID-19 pandemic exacerbated these inequalities between EMCs and the white majority, with higher infection and death rates recorded [18]. The UK government’s response to the pandemic included wide-scale Infection Prevention and Control (IPC) messaging focused on staying home, wearing masks, social distancing and COVID-19 vaccination [19]. During this time, language barriers [20], historical social and economic injustices [21], messages that clashed with cultural values and underrepresentation of ethnic minority voices [22], may have led to low uptake and compliance with IPC measures [21, 23]. As further evidence emerged showing the negative impacts of the lockdown and other restrictions on health behaviours including diet and PA [24–27], public health messaging began to promote healthy behaviours. It is possible, however, that many in EMCs received this messaging with the same distrust as they had received previous IPC messaging [20, 28].

Although, promoting public health requires a whole systems approach [29, 30], health promotion messages when appropriately designed have proven useful as one of the whole systems approach [31, 32] especially at the individual level useful in promoting health behaviour change [33–35]. Multiple reports have highlighted the need for tailored public health messaging to underserved groups, including EMCs [20] to ensure greater accessibility and positive behaviour change. The call has been for messaging that is simple, available in minority languages, co-produced, consistent with cultural norms and publicised through local community channels [36]. These suggestions are rarely applied, however, to messaging to support members of EMCs to eat more healthily and be more active. For such messaging to be effective, it is important to understand how these underserved communities currently receive public health messaging and their perceptions of how messaging is shaped.

Most of the evidence of the impact of public health communication on the behaviour of EMCs [20, 36] during the pandemic is limited to the impact of IPC messaging. Evidence of the acceptability and reception of PA and healthy eating messaging is scarce. A recent government review of messaging during the pandemic [20] highlighted the need for qualitative evidence to understand how messaging was received and perceived by these communities.

This paper describes: 1) a scoping review undertaken to identify the different categories of PA and healthy eating messages produced during the pandemic that were targeted specifically towards EMCs in preparation for a subsequent qualitative study; and 2) a qualitative study aimed to understand how these different categories of PA and healthy eating messages were seen, heard, and received by EMCs.

This study was conducted as part of a UKRI-ESRC funded project entitled: *Consortium on Practices for Wellbeing and Resilience in BAME Families and Communities (Co-POWeR)*, within the Work Package on Physical Activity and Nutrition (WP4).

## Methods

### Ethics statement

This study was conducted according to the guidelines laid down in the Declaration of Helsinki and all procedures involving research study participants were approved by the AREA Research Ethics Committee (no. 20–120), University of Leeds and the Faculty of Environmental and Life Sciences Ethics Committee (no. 65351.A1), University of Southampton. Written informed consent was obtained from all participants.

### Study design

A combination of scoping review and qualitative research methods were used to address research objectives. Community engagement was embedded in the study from start to finish. People from EMCs were engaged in developing the scoping review search strategy, reviewing, and refining the interview topic guides, and contributing to all decision-making process on the study (four CE partners were members of the core project team and attended project planning meetings). A CE partner also contributed to data extraction and the draft manuscript was reviewed by an additional CE partner to ensure genuine reflection of EMCs realities.

### Scoping review

#### Rationale

Addressing the first study aim, a scoping review was chosen over a systematic review to identify the different types of messaging available and allow for inclusion of evidence from a wide variety of sources [37, 38]. The review aimed to include all data sources, including journal articles, containing messaging. Messaging was taken to mean any information developed for the public to promote PA and healthy eating. The review followed the 5-step methodological framework by Arksey and O’Malley [37]; refined using the Joanna Briggs Institute (JBI) framework for scoping reviews [39], to ensure a critical review of literature from both research and non-research sources. A decision was taken to also include Community Engagement (CE) to increase the robustness of the methodology. The Preferred Reporting Items for Systematic Reviews and Meta-Analyses extension for Scoping Reviews (PRISMA-ScR) checklist [40] was followed (S1 Checklist).

#### Search strategy

An initial scoping search was conducted using Google Scholar, MEDLINE, NICE Evidence search, Cochrane reviews and JBI Evidence Synthesis to inform the search strategy. The search strategy included keywords and synonyms related to: 1) physical activity; 2) healthy eating; 3) messaging; and 4) ethnic minorities.

The UK Office for National Statistics (ONS) definition of ethnic minorities includes every ethnic group except the White British group. This ONS definition of ethnic minorities was adopted for use during the scoping review.

Searches of both scientific databases and grey literature were conducted.

MEDLINE, CINAHL and Scopus were searched for PA and healthy eating messaging targeted towards EMCs produced between December 2019 and 30^th^ September 2021 (S1 Text). This start date of December 2019 coincides with the start date of the COVID-19 pandemic in the UK. Although, it is unlikely considering the length of time it takes to produce and disseminate public health messaging, that public health messaging would have been available at that time, the date was chosen to ensure no messaging was missed. The end date of the search was also chosen to coincide with the start of data collection during the qualitative study. This was to ensure that participants involved in the qualitative study could reflect during data collection on messaging that was available to them at that point during the pandemic.

To identify grey literature and non-research sources, various websites and platforms (S1 Table) were searched using a combination of keywords and appropriate syntax where supported. Public members from EMCs were also engaged through CE activities to identify their sources of healthy eating and PA information, and those were searched. Finally, a hand search of the reference lists of key studies was conducted.

We did not carry out a search in other languages than English, because much of the public health messaging available in other languages would often have an English version which we would have picked up in the search and which we included in the review. The search strategy was developed with input from a librarian and a team of experts. This search was not conducted to be a comprehensive search of all messaging available but to scope the different categories of messaging available during the specific period of the COVID-19 pandemic, hence the search strategy.

#### Study selection

EndNote (version 20) was used to remove duplicates before transferring the scientific search results (n = 802 publications) to Rayyan [41], a web app for systematic reviews, where study selection was performed in two stages: 1) title and abstracts screening; and 2) full-text screening. Two independent reviewers (OG, BM-S) reviewed each result against the inclusion and exclusion criteria in Table 1 and a third reviewer (SA) was available to resolve disagreements.

**Table 1:** Inclusion and exclusion criteria for scoping review.

Group	Inclusion Criteria	Exclusion Criteria
Content	Contains messages, guidelines or information targeted to the **members of the public from ethnic minority background by having at least one of the following:** a) Mentions BAME or other similar words like “ethnic minority”, “immigrants”, “BME” etc. b) Targets at least one BAME group: for example, a paper targeted to only Asians would still be included even though, it doesn’t address Black people c) Have options to view translated versions or copies in a different language	Messages, guidelines or information not targeted to ethnic minority populations
	Contains messages relevant to physical activity or/and healthy eating	Messages, guidelines or information not targeted to the public e.g., messages to the research community, athletes/sport elites or to the government.
Date	Made publicly available between December 2019 and September, 2021	
Age	Relevant to all or any age group including children, adults or the elderly.	
Population setting	Relevant to the UK population^a^ by having at least one of the following: a) Written by UK authors or a UK based organization b) Identifies the UK as a target population c) Made use of UK data d) Produced by regional and international authorities like the UN agencies and hence targeted to a global or regional population.	Messages not applicable to the UK population
Language	Any language	
Design or literature type	Peer reviewed Scientific articles, government policies, guidance and reports^b^	Social media posts, blog posts and media articles made by the people

^a^Unsure papers added to a maybe list for one-on-one decision by reviewers at an agreement meeting.

^b^The aim is to limit the review to literature produced at the top level of government and authority rather than literature produced by the people (social media, personal blogs, etc.)

For grey literature results, screening was performed using a combination of Rayyan and Microsoft Excel (version 2022). Full text screening was conducted independently by two reviewers (OG, SA), with the third reviewer (BM-S) resolving disagreements.

Papers and reports were included if they contained PA and/or healthy eating advice targeting UK EMCs, with types of literature restricted to scientific articles, government policies, guidance and reports, and content produced by key national stakeholders in public health. Social media content, blog posts and media articles were excluded. Papers published in languages other than English were included in the search. Two papers not written in English were excluded as they did not meet inclusion criteria. Reviewers piloted screening with 10 records to ensure mutual understanding of inclusion/exclusion criteria.

#### Data extraction and analysis

Author, title, literature type, aims, date and messages promoting healthy eating and/or PA were extracted into Microsoft Excel by OG and 20% of the extracted data were checked by BM-S and SA independently to ensure reliability and reproducibility. The extracted data were then transferred to NViVo as externals, where the data were grouped into categories of PA and healthy eating messages.

### Group and individual interviews

Group and individual interviews were used to address the second study aim, based on participants’ preferences.

#### Study participants and recruitment strategy

People from EMCs aged >15 years, living in the UK during the COVID-19 pandemic were eligible to take part. For this qualitative study, we used the definition of ethnic minorities that had been used in the Co-POWeR project; this included people belonging to any UK ONS high-level ethnic group except the White ethnic group. Care was taken to ensure group interviews were run with participants of similar ages to aid openness. A variety of means was adopted to facilitate the recruitment of an ethnically diverse group of participants. This included: 1) face-to-face visits by researchers to community groups and locations resulting in snowballing to other community groups; 2) Social media recruitment using project posters and 3) Snowballing. The total number of people approached is unknown due to the recruitment approach adopted. Of the 43 people recruited, two who originally consented to take part were no longer available at the time of data collection. A study information sheet was provided to all participants and study details were explained during a phone call, Zoom call or face-to-face by a researcher (OG). This approach served to establish a relationship with potential participants before data collection. Written and verbal informed consent was obtained from all participants prior to data collection. Participants were given a voucher at the end of the study, as a token of appreciation for their time.

#### Data collection

A topic guide was developed with project collaborators and CE partners involved in the wider Co-POWeR WP4 study. Data on which this paper is based are from the following questions: 1) What general messages have you seen that promotes increase in PA and healthy diet during COVID-19?

1. Where did you find the information?
2. Where do you look for these kinds of information?
3. What did you think of this information?
4. Do they make you want to be more active or eat healthy?

A pilot group interview was run on the 29^th^ of July 2021, after which a total of 10 group interviews (2 to 6 people per group) and five one-to-one interviews lasting between 60–90 minutes were conducted between the 29^th^ of July 2021 and the 8^th^ of March 2022. Some participant groups, including young people and older people (four group and an individual interview) specifically requested face-to-face data collection. Face-to-face interviews were held at agreed locations (a community group venue, a food bank and on a university campus) with COVID-19 safety procedures duly followed. All face-to-face group interviews were led by OG and had the community co-ordinator for the group present to foster open communication. The rest of the interviews were conducted online using Zoom conferencing software; facilitated by OG and co-moderated by MS, SA and BS. All had previous experience of conducting qualitative interviews and CE with people from EMCs, being from these communities themselves.

All interviews were audio-recorded using either Zoom recording or a digital recorder and transcribed by a professional service with a confidentiality agreement in place. The lead researcher (OG) checked the transcripts to ensure accuracy and that non-verbal communication and accent differences were captured. The transcripts were fully anonymised before analysis begun and participants were given an alphanumeric identifier starting with “I” or “G”; indicating individual interview or group interview, followed by a serial number.

#### Data analysis

The six stages of thematic analysis proposed by Braun and Clarke [42] were followed in the inductive analysis of transcripts: data familiarisation, initial code generation, searching for themes, reviewing themes, defining, and naming themes and producing the report. The analysis was carried out using NViVo-12 software independently by OG and SA, after which coding was compared and any disagreements resolved through discussion. The wider research team reviewed and agreed on key themes, sub-themes and illustrative quotes to be used. The Consolidated Criteria for Reporting Qualitative Research (COREQ) was followed in producing this manuscript.

## Results

### Scoping review

#### Identified studies

**Fig 1:**
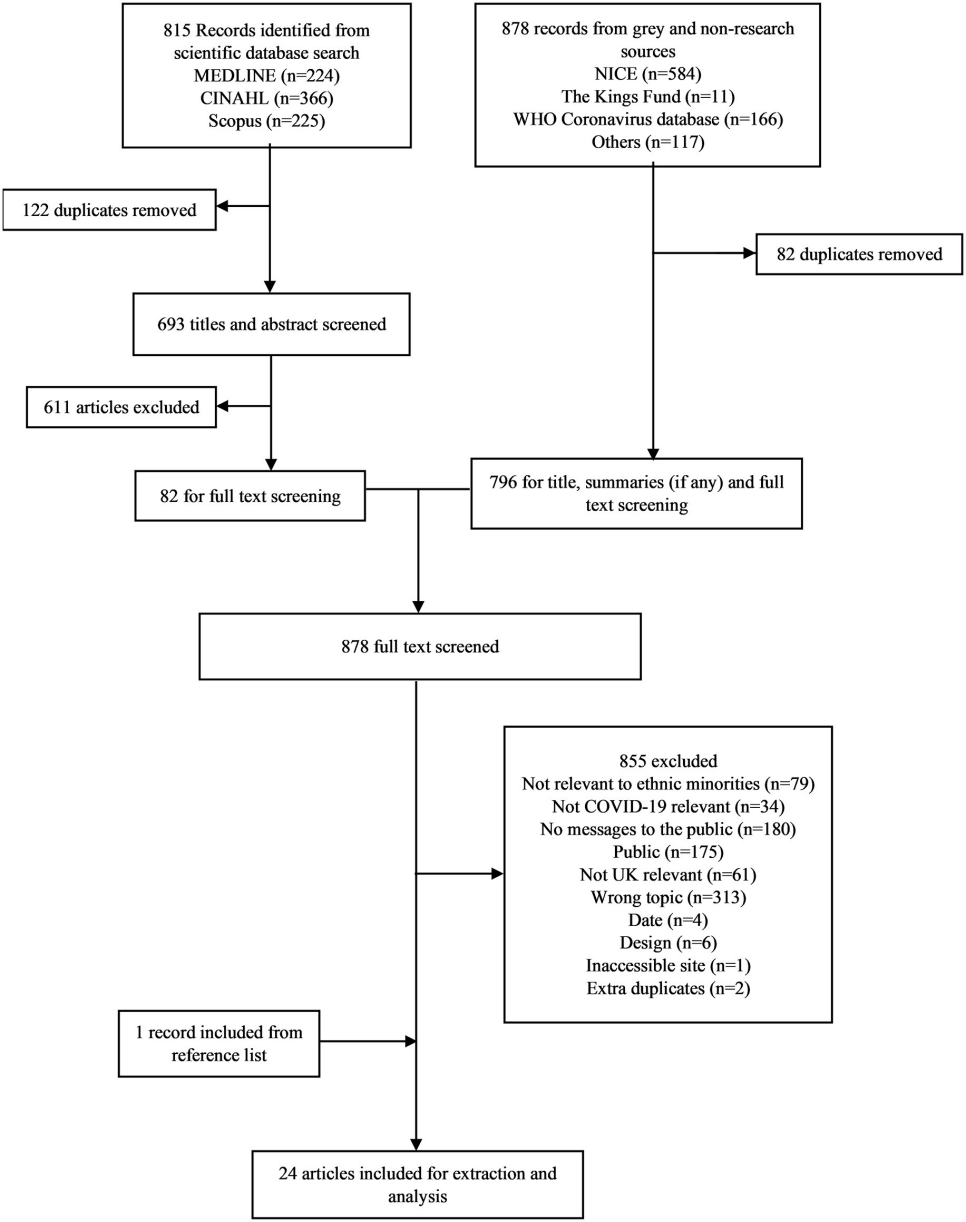
PRISMA flow diagram of study selection process.

From 815 records identified by the scientific database search, 122 duplicates were removed, and 611 articles were excluded during title and abstract screening, the latter with 88% interrater agreement, leaving 82 full texts as in Fig 1. The grey literature search identified an additional 878 records, from which 82 duplicates were removed, leaving 796 records for full text screening. A total of 878 records (82 full texts from the scientific search and 796 from the grey search) were screened and 855 records were excluded. One record was added after a hand search of references and a total of 24 records were included in the review.

Included records comprised three journal articles, two reviews, 15 website articles, some of which included images, three digital documents/booklet and one poster. The records with publication dates were published between March 2020 and September 2021. Five records which were articles on public websites had been further updated by the time of extraction and so were dated December 2021. Eleven records included information relevant to both PA and healthy eating, four referred only to healthy eating and nine only PA. Included records and their characteristics are presented in Table 2.

**Table 2:** Included records and their characteristics.

No	Title	Authors	Date	Aim of Paper	Methods/Paper type
1	Helping people get fit to fight COVID [43]	NHS Kent and Medway	Unspecified	Provide information to help people remain fit and healthy to limit the impact of COVID	Website article
2	Covid 19: Vitamin D and BAME populations [44]	Gloucestershire County Council	Unspecified	No clear aim indicated. To address queries on the role of Vitamin D in different COVID-19 outcomes	Web document
3	Children and Young People’s Mental Health and Coronavirus (COVID-19); A booklet for Black, Asian and Minority Ethnic (BAME) parents from stem4 [45]	Dr Nihara Krause for stem4 charity	2020	Providing guidance and advice to parents especially BAME, on how to support the mental health of children and young people during COVID and beyond	Digital booklet
4	Covid-19 Lifestyle Advice for BAME community [46]	National forum for health and wellbeing and British Association of Physicians of Indian Origin, Indian association Manchester	October 2020	Poster produced to explain what can be done and how to look after health both during the pandemic and in general. Overall aim is to help everyone adapt healthy lifestyle and lead a healthy life.	Posters
5	Coronavirus infection and pregnancy—information for pregnant women and their families [47]	Royal College of Obstetricians & Gynaecologists	Unspecified	To answer frequently asked questions on COVID-19 and pregnancy	Online website article
6	Latest COVID19 Advice for British Muslims [48]	The Muslim Council of Britain	February 2020	COVID 19 advice for Muslims.	Website with extra resources to further government guidance
7	What factors put you at risk from coronavirus? [49]	British Heart Foundation	2020	To explain the kinds of factors that affect risk from coronavirus and what can be done about them to reduce risk	Website article for public
8	Having a healthy Ramadan at home [50]	British Nutrition Foundation	Apr-20	To provide practical nutrition advice to help Muslims stay healthy, while following government guidelines in relation to coronavirus	Online web article
9	Vitamin D for COVID-19 and Acute Respiratory Tract Infections; SACN and NICE review the evidence [51]	British Nutrition Foundation	Jul-20	To summarize the rapid review of the evidence on Vitamin D and COVID-19 and Acute respiratory tract infections done by NICE and SACN	Online web article
10	Keeping safe in your home during coronavirus [52]	Leeds City council	15-Dec-20	How to keep those you live with safe during COVID-19. Guidance for households with grandparents, parents and children living together	Digital booklet
11	Keeping well at home [53]	The Healthy Ageing Research Group and Manchester Institute for Collaborative Research on Ageing	08-Jun-20	Providing lots of ideas and suggestions to help us keep well. It is written for those with less or no access to online resources	Digital booklet
12	Coronavirus and your wellbeing—for young people [54]	MIND	21-Dec-21	To provide information for young people on looking after wellbeing during coronavirus	Coronavirus and your wellbeing—for young people
13	Coronavirus and mental health tips [55]	Mental health foundation	15-Dec-21	To provide tips that will help people look after their mental health while staying at home	Website article for public
14	Mental health advice for older people during the coronavirus outbreak [56]	Mental health foundation	December 2021	To provide advice to help older people look after their mental health	Website article for public
15	Coping with coronavirus: a guide for young people [57]	The Mental health foundation, MHF Young leaders and Leaders Unlocked	December 2021	To provide tips for young people on how to cope with the coronavirus	Website article
16	Getting through COVID-19: tips from a key worker [58]	Mental health foundation	December 2021	Suba is a doctor, humanitarian and podcaster who has shared her five top tips for maintaining good mental health as a key worker during the pandemic.	Website article
17	Vitamin D and coronavirus: is there evidence it can help? [59]	Natalie Healey, reviewed by Dr Sarah Jarvis MBE for patient	May 2020	To provide clarity on the link between Vitamin D and COVID-19 risks and advice on the need to take supplements during the lockdown	Website article
18	Why are Black and Asian people at greater risk from COVID-19? [60]	Ellie Broughton, reviewed by Dr Sarah Jarvis MBE for patient	July 2020	To provide advice on understanding how and why the virus is impacting people of different ethnicities in different ways	Website article
19	Look after your mental health and wellbeing when staying at home [61]	Mental Health Foundation	Unspecified	Providing tips to people to help them look after their mental health	Website article
20	Healthwise, Part 2. Eating a balanced diet [62]	Linda Nazarko	June 2021	To explore how readers can remain healthy and well by eating a healthy, balanced diet during the COVID-19 pandemic	Research article
21	Coronavirus and children and young people’s mental health—Emerging evidence, Issue 1 [63]	Melissa A Cortina, Anna Gilleard and Jess Deighton.	May 2020	A rapid review of evidence from around the world that answers three main questions: 1) What are the key mental health challenges for children and young people during the coronavirus pandemic? 2) Are there any particular vulnerable groups? 3) What might help children and young people manage these challenges?	Rapid review report online
22	Covid-19 lockdown: Ethnic differences in children’s self-reported physical activity and the importance of leaving the home environment; a longitudinal and cross-sectional study from the Born in Bradford birth cohort study [64]	Daniel D. Bingham, Andy Daly-Smith, Jennifer Hall et al.	September 2021	The study aims to: 1) report children’s self-reported physical activity (PA) during the first COVID-19 UK lockdown and identify associated factors; 2) examine changes in children’s self-reported PA prior to and during the first UK lockdown	Research article–longitudinal and cross-sectional study
23	Balancing Immune System [65]	James Paul Pandarakalam	December 2020	To examine the possible factors responsible for the suppressed general immunity among the BAME population	Research article
24	Emerging Evidence: Coronavirus and children and young people’s mental health -Issue 8 [66]	Mairi Jeffery, Tanya Lereya, Julian Edbrooke-Childs et al.	June 2021	The last issue of a series of 8 rapid reviews on coronavirus and children and young people’s mental health. This final concluding issue aims to reiterate what has been learnt from the reviews of the literature, emphasising some of the key studies and setting out recommendations for supporting children and young people’s mental health as the pandemic continues and beyond	Summary and report of a rapid review

All 24 records included in this review gave generic information on PA and/or healthy eating and used words related to ethnic minority groups at least once in their text in line with the inclusion criteria. However, very little of the messaging within the records was specifically targeted to EMCs. For example, a record could be a 5-page document on healthy eating advice but only include one sentence about ethnic minorities.

Three main categories summarised the included messages: PA, healthy eating and risks.

#### Category 1: Physical activity

This category included messaging encouraging people to be active, its benefits and recommendations. People were encouraged to be active around the home or engage in activities they enjoy such as walking or dancing. The two main benefits of PA highlighted were better mental health and wellbeing [45, 48, 65] and improved immune function [53, 65]. Other benefits mentioned included prevention of long-term conditions [53], weight management [53] and improved balance and energy [53, 65]. The importance of PA in pregnancy [47] was mentioned and one record targeted older adults and provided detailed information on safety measures during PA to prevent falls and injuries [53]. One record also mentioned the WHO recommendations for PA for adults [46] while another stressed daily PA and promoted brief, consistent activity as beneficial [53].

#### Category 2: Healthy eating

This category described messaging encouraging people to eat healthily, what foods were healthy and their benefits.

These records stressed the main benefit of healthy eating was to improve immune function to prevent COVID-19 infection and other diseases [53, 65]. Specific nutrients like Vitamins C and E, folate, beta-carotene and zinc as well as specific food groups like fruit and vegetables, and foods rich in omega-3 fatty acids were highlighted for their immune regulating properties. People were encouraged to drink water and cut down on processed and sugary foods and drinks [50, 53, 62, 65]. Tips to ensure healthy eating despite the lockdown included planning food shopping to avoid waste [50, 53, 62], going for dried foods as they have longer shelf-lives [50, 62] and adopting healthy cooking methods [50, 62].

Records also referred readers to healthy eating recommendations such as the UK Eatwell guide [50, 62] and the Asian healthy eating pyramid [46]. Only one record explained portion sizes for fruit and vegetables [62]. A myth about the use of alcohol to kill the coronavirus was refuted, and information on the negative effects of alcohol consumption was provided including its associations with long term conditions, reduced immune function and consequently increased COVID-19 risk [65]. Alcohol consumers were encouraged to do so in moderation up to 14 units spaced across a week [46, 53]. Nutrition advice during Ramadan fasting was provided by one record [50].

#### Category 3: Risk

Compared to the other categories, this category included more targeted messaging for EMCs. Messaging focused on the higher risks of infection and death from coronavirus amongst EMCs and how PA and healthy diet could reduce this risk [43, 46, 47, 49, 60, 65].

Many of the risk messages were focused on Vitamin D. It was explained that people from ethnic minorities were more prone to vitamin D deficiency because higher melanin levels in their skin resulted in reduced Vitamin D production in the skin [44, 65]. Although unclear and not well-evidenced, links between Vitamin D and COVID-19 risk in EMCs were mentioned in some records [44, 59], as was the link between sufficient Vitamin D and a healthy immune system [44, 65]. Most of this messaging was focused on advising EMCs to take Vitamin D supplements [46, 51, 59, 60, 65]. Oily fish, eggs and fortified breakfast cereals were also mentioned to be good sources of Vitamin D [59, 65].

The importance of having a healthy weight for optimal immune functioning was highlighted by one record explaining that fat cells suppress immune function [65]. Another record shared information on what a healthy Body Mass Index, waist-hip ratio and waist circumference were [46]. Switching to a low-carbohydrate diet or total elimination of carbohydrates as a weight loss technique was advised against and people were encouraged to adopt healthier ways of losing weight [49, 62].

### Qualitative study

A total of 41 participants (21 women) between 18–86 years old took part in the focus groups and interviews (Table 3). Over half of participants (68%) identified as Black, Black British, African and Caribbean according to the ONS 2021 ethnicity classification, while the other 32% were from Asian, Mixed and other EMCs. Participants were mostly resident in England (63%).

**Table 3:** Participants’ characteristics.

Age group (years)	Number of participants (%)
18–19 (Teenagers)	8 (20)
20–34 (Young adults)	12 (29)
35–64 (Adults)	18 (44)
≥ 65 (Older adults)	3 (7)
**Gender**	
Men	20 (49)
Women	21 (51)
**Ethnicity**	
Asian or Asian British	4 (10)
Black, Black British, Caribbean or African	28 (68)
Mixed or multiple ethnic groups	4 (10)
Other ethnic groups	5 (12)
**Country of Residence**	
England	26 (63.4)
Wales	14 (34.2)
Scotland	1 (2.4)

Five main themes were identified that described the data: 1) lack of awareness of messaging; 2) responses to PA messages; 3) responses to healthy eating messages; 4) perceptions of risk messaging; and 5) perceptions of conflict in messages. An underlying theme of trust in the messaging was observed across all themes.

Most participants in all age groups and ethnicities, either had not seen or could not remember seeing any PA and healthy eating messaging during the COVID-19 pandemic. Those who had seen messages attributed these to sources such as social media, and ethnically diverse community groups to which they belonged to. Most messages seen were on PA; only a few participants acknowledged seeing messaging on healthy eating which was mostly focused on the benefits of healthy eating for improved immune function. Participants also spoke about how messaging suggested an inherent COVID-19 risk among EMCs. Some described how, over time, they had become aware that the higher infection and death rates were not inherent but due to other factors such as increased exposure to the virus as frontline workers. Finally, some participants especially teenagers and young adults spoke about their perceptions of government messaging being conflicting and how this reduced their trust in the government and messaging. For example, government messaging to stay healthy was not reflected in the government’s choice to re-open fast-food restaurants while gyms remained close. Table 4 provides a detailed summary of these themes, subthemes and illustrative quotes.

**Table 4:** Detailed themes, subthemes and illustrative quotes from qualitative study.

Theme	Sub-themes	Explanation of themes	Example Quotes
Lack of awareness of messaging	Personal reasons (prior negative experiences resulting in distrust, social circle, geographical location)	Some participants explained they avoided seeking such messages due to previous negative experiences with government COVID-19 information. This led to a lack of trust in the government and its messaging.	“I just thought they were contradicting themselves. Masks. No masks…I thought they knew what they were doing…I just did not trust. I lost my trust in them” I16, adult “You know I told you I have stopped watching the news ‘cos it puts so much fear in me.” I40, adult
		Some adults also attributed this to their social circle by saying that they just did not watch enough TV to see messages or that their friends just did not talk about these things.	“I don’t think that message is out there, and then again, maybe my circle of friends.” I38, adult “Mainly because I didn’t really watch much television. It was more Netflix, so I didn’t see any adverts” I41, adult.
		Only one adult woman attributed this lack of messaging specifically to their city of residence.	“[City withheld] needs an upgrade cause they are focusing on other things, so we are left behind at the moment”. I13, adult
	Lack of government messaging	Mostly teenagers and young adults emphasised they did not come across messages because public health priorities were focused elsewhere.	“Not much has been put forward in regard to physical activities and eating right, because I think the government were busy fighting, closing borders and putting people on furlough” G02, young adult. “The news wasn’t talking about people sorting out their health, they were mostly talking about when are we getting the vaccine… instead of focusing on what a person can do today…” G23, teenager
	Delay in producing and disseminating relevant government messaging	Members of a community youth group spoke about the insufficient priority placed on translating messages.	“I think they delayed in our communities–especially the languages, because they said it in English. Half of our community do not even understand English. Most of them only understand our first language”. Community group Leader, Adult
		Delays affected well-meaning efforts to increase reach of messaging making it harder for accurate messaging to be accepted once misinformation has been propagated.	“We were trying to find the information ourselves, where someone may be finding the wrong information. So, the first time someone hears something that’s what they’re going to think and you’re going to have to convince them afterwards” G23, Teenager
		Efforts to reach targeted community and religious groups affected by the delays in dissemination.	“They…started targeting the mosques to reach the community but at the same time the mosques were closed so how could they do that”. Community group leader, Adult
Responses to PA messaging	Sources of PA messaging	Participants (mainly adults regardless of ethnicity and country of residence) reported their main sources to be the news, parliamentary briefings and television.	“But the parliament news, that’s where you get the real thing, they tell you to try and do the exercise on a daily basis” G33, adult
		Young people reported social media and other digital platforms as their main source.	“They weren’t from the government though; they were just general people that were fit and were trying to get people to understand why they should get fit. Generally normal people…Nothing from people who were actually meant to help us.” G23 and G22, teenagers.
		Specific examples reported across all age groups were YouTube channels, especially content created by Joe Wicks, and podcasts.	“I don’t know if you know Joe Wicks, this exercise guy on YouTube, he’s a fitness coach. I feel like he did more for promoting physical activity during the lockdown than the government really did… he made it fun and exciting… He was targeted at kids, adults, anybody of any age. It was very much accessible to everybody. Why isn’t the government working more with Joe Wicks to change the language and to change the mindset towards exercising at home during the pandemic?” G01, young adult woman.
		All age groups identified community groups and social networks as key sources.	“He is our community leader. he’s keeping us active, if it wasn’t him, we will all stay the same, now we are keeping active and it is helping our mental health” G21, teenager
		Parents and guardians with children reported school homework to be their main, and in some cases, only source.	“…I think what brought more exercise to me was because of the children. In his school they were always directing us to…a PE teacher that became very popular… So, I think that just made me realise that ok, this exercise thing is good, but apart from that, I don’t remember seeing any adverts” I14, Adult.
	Perceptions of Government guidelines during lockdown	Government guidelines on outdoor physical activity engagement was perceived to be important by mainly adult participants.	“…I know that in the news they kept saying you can go out and do your exercise. Though we weren’t allowed to go out, you could go out and exercise so, I think that showed how important exercise was” I38, adult
		Teenagers and young adults believed the messages were not encouraging.	“No, the government were just like, oh, please, just do one small hour and then go back inside.” G03, Young adult.
Responses to healthy eating messages	Sources	Digital sources such as YouTube and other social media platforms.	“There was a lot about it especially on social media…Some people were trying to motivate people to eat healthily” G27, teenager.
		Community groups, youth activity clubs and their coaches.	“…They used to teach us this in the boxing club. During the pandemic, I used to have elderly people in my family, and I see the community leader and group come with good bags of food, some vegetable, he used to bring it to families around the docks” G21, teenager.
	Messages on benefits of healthy eating	Improved immune function to fight against the virus.	“They did say during the lockdown…we have to eat well because when you eat well your immunity is stronger… I would say to some extent that yes, it has moved us to look at what we eat especially sugar–to reduce our sugar intake. since covid happened it has given us the impetus to move a lot more towards a plant-based diet.” I38, Adult.
		Perceived benefits of Vitamin D for improving immunity.	“I know that there was a research that was carried out. I think a lot of hospitals were doing it. Vitamin D helps with the immune system, and it helps in fighting, so I don’t know how solid that research was, but a lot of health workers were taught to take Vitamin D.” G02, Young adult.
Perceptions of risk messaging	Generic risk messages	Risk messages focused on staying at home, wearing masks, getting vaccinated and infection and death updates.	“The only public health messages that I came in contact with was like on the billboards. It will be like, stay at home. If you go out, you’ll kill somebody or something like that. You know, the very, very strong emphasis on staying at home, which is good, you know” G03, Young adult.
	Inherent COVID-19 risk in EMCs	Participants from Black and mixed ethnicities raised concerns that inherent risk messaging led to increased discriminatory behaviour.	“I think we were treated in a different way because people were thinking that our skin, our people, like African people were getting this virus worse than others… we were the one that people can point their fingers at…they are dying too much, maybe because they don’t believe, they have too much faith, so they don’t protect themselves, maybe they have lack of knowledge… So, it’s like, they were treating us in a different way” I39, adult.
	Emerging risk factors	Participants now perceive other risk factors such as housing arrangements, work conditions and exposure to the virus to be responsible for higher COVID-19 infection and deaths.	“I mean after 20 months or more now, we now know that minority people are not dying because of their ethnicity. It is because they were probably more exposed to it. Because in Africa (country withheld) where I come from, they are not as strict, social distancing is almost non-existent and people are not dying there as much. So, there must be a reason why minority people here died as much as they did.” I38, adult “I think it is generally the kind of jobs that BAME people do that exposes them to the virus more…” I19, adult.
Perceptions of conflict in messaging		Teenagers and young adults perceived government messaging to be contradictory to their corresponding actions making them feel confused.	“Every time Boris Johnson would come and speak, he would speak and then they would put up [a fast-food brand mentioned] is open, we’re going to open xxx on this day, xxx on this day. So basically, they were pushing you not to train and to be unhealthy. Then the same person would come and tell you oh yes, you need to run, you need to train for at least an hour per day, but xxx is open, why couldn’t they open the gyms? That would motivate more people as they would be like, there’s no fast food so I’ll just go to the gym and get my workout in and see how I feel” G27, teenager. “I found the messages by the government were very antagonistic. Like, okay, make sure you’re running where there’s lots of air. Stay away from anybody you don’t know. If you have to run wear a mask. Do it in the early hours. You know, I know they didn’t say things like that, I’m just over exaggerating, but that was kind of the vibe and the tone that you get from the message. It was very much like, you know, keep away from everybody and unless you really have to, don’t do it”. G01, young adult. “The same amount of bigger vigour or vehemency is not given to, stay healthy, keep your mind active, go do physical activity” G03, young adult.
		Refusal to consider subsequent government messaging as a result of contradictions and lack of trust.	“Personally, I never paid attention to that [referring to messages from the government] because to me it doesn’t make any sense. Now, they brought out the vaccines and they tell you that you have to take two vaccines to be able to avoid getting covid but when you get the vaccines they tell you, you’re still able to catch covid…At one point, I stopped listening to all of it”. G27, teenager

## Discussion

This mixed-methods scoping review and qualitative study identified the PA and healthy eating messages targeted at EMCs during the COVID-19 pandemic of 2020 to 2021 and explored how these communities received such messages. Analysis showed that: 1) public health messaging specifically targeted to EMCs to improve PA and healthy eating was limited; and 2) this limited messaging was either entirely missed by these communities or, where received, was not interpreted as intended.

### What were the main categories PA and healthy eating messaging targeting EMCs?

During the COVID-19 pandemic, there was an enormous amount of public messaging mainly focused on IPC associated behaviours [67], but also on other health behaviours including PA and healthy eating [68]. The findings of the present study suggest that amongst this plethora of messaging, there was very little that specifically targeted EMCs. This is in line with previous studies [20, 28, 36, 69, 70] that found most government messaging not to be targeted or culturally appropriate and while some guidance was translated, the release was slow and usually difficult to access [70]. These reports have, however, been focused on COVID-19 IPC and vaccination messaging. To the best of the authors’ knowledge, there has been no previous review of the PA or healthy eating messaging targeting EMCs during the pandemic.

The available PA and healthy eating messaging either presented the benefits of, and recommendations for, being active and eating healthily or focused on informing EMCs of their vulnerability to COVID-19 infection. There were difficulties in defining what targeted messaging was as all records included in the review were in English and only a few explicitly identified EMCs as their target. Although all 24 papers included words relating to EMCs, only COVID-19 risk-related information tended to be specific to these communities. Most of the PA and healthy eating messages addressed the general population. Studies have shown when PA and healthy eating messages are not tailored and targeted, messages will not necessarily be relevant to or take account of the needs of minority communities and therefore risk accentuating existing inequalities [71, 72]. The importance of tailoring messaging to EMCs was highlighted in an evidence summary published by the Scientific Advice Group for Emergencies in September 2020 [36]. This group proved that culturally tailored messaging increased accessibility and acceptability of COVID-19 IPC messages to EMCs. Conversely, targeting messaging to EMCs may also have disadvantages, such as possibly being perceived as stigmatising [36] and if not made specific, may ignore important cultural and other differences between minority communities. Care needs to be taken to avoid these negative impacts.

### How was PA and healthy eating messaging received by EMCs?

The lack of targeted messaging on PA and healthy eating was confirmed in the qualitative data collected during this study. Most participants reported that they had not come across any messaging on PA and healthy eating at all during the pandemic.

There are several possible explanations for this perception, all of which relate in some way to EMCs levels of trust in the UK government. As some participants said, previous negative experiences with government and public health messaging at the start of the pandemic [20], led to distrust and avoidance of subsequent messaging. This phenomenon was also observed in a study of young people of diverse ethnicities in Southampton, London and Edinburgh [22], where some young people avoided specific sources of government information and messaging because they perceived it as negative and anxiety-causing, and because they no longer trusted the government’s handling of the pandemic [22]. This may have also led to people moving away from government sources of information to more trusted community-based sources. For example, participants in the present study who did come across PA and healthy eating messaging emphasised that most of this came through groups in their communities and on social media. These community groups tended to be age-specific and include people from diverse ethnicities. It is well-established that some ethnic minorities have greater trust in their communities and religious leaders than in more anonymous government institutions, and so prefer to receive messaging from them [36, 73]. This is despite the fact that people from diverse communities acknowledge the government as a source of official information [22].

The sources of PA and healthy eating messaging accessed was a focus for participants’ reflections on how the UK government failed to address the needs EMCs during the pandemic. Some participants, especially young people, expressed dissatisfaction with the lack of health relevant messaging on PA and healthy eating by the government and public health authorities “*who were meant to help us*”; leaving them to rely on social media and other sources for information. They suggested that the government partner with content creators who already had the following to promote PA and healthy eating. Participants also expressed frustration at the fact that the focus of messaging for ethnic minorities was on COVID-19 IPC measures and vaccination, with an apparent disregard of other health relevant behaviours. Despite low trust of the authorities by EMCs, there is still an expectation for the government to be the key source for reliable and accurate messaging. There is a contradiction apparent in this. It could be, as has been observed before [74], that availability of better messaging may facilitate and rebuild trust in the government addressing the disillusionment that many EMCs experienced during COVID. It may also be, however, that effort needs first to be put into rebuilding trust before it is worth additional government campaigns to promote PA and health eating in EMCs. One positive step would be a genuine commitment to co-production of these message [36, 74, 75] followed by co-dissemination of messaging with community and religious groups as was advised in the present study There is some evidence that this would facilitate increased acceptance of public health messaging [36, 74, 75].

Social media was another key source of information during the pandemic, to the point where the WHO described it as an infodemic [76]. While some social media messages were accurate, much of it was misinformation, myths and disinformation, deliberately sponsored to prevent people from complying with guidelines; this was seen particularly in anti-vaccine messaging [76]. Study participants felt that the delay in government messaging reaching their communities led them to resort to seeking information from other sources which may have been less accurate. If mis- and disinformation is first heard, it may become difficult for people to accept later, more accurate messaging; the first source of messaging is likely to be the most trusted [77].

In line with some previous studies, participants interpreted some of the risk messages as finger-pointing, indicating that there was something inherently wrong with their communities, therefore, being racially discriminatory [73, 74]. Both minority and white ethnicity participants in a qualitative study in England and Wales in 2020, expressed their displeasure at the framing of messaging on the ethnic inequalities in COVID-19 risk and how it contributed to further inequalities and obscured wider structural inequalities present in the country [74]. The same was expressed by community leaders from EMCs in a qualitative study in the West Midlands [73]. Messaging perceived as discriminatory and racist is likely to provoke a defensive response, which might in turn lead people to be unreceptive to subsequent messaging. For example, most of the COVID-19 risk messaging seen identified in the present scoping review was centred on Vitamin D, its role in improving immune function and the potential role in reducing risks of either COVID-19 infection or its severity. However, only one participant in our qualitative study had actually seen any information on the Vitamin D-COVID link and this was because of their role as a health worker. It is possible that Vitamin D messaging, like other risk messaging, was perceived as racist and was less likely to be heard by EMCs; they may have already stopped listening to any government messaging at this point. There is a tension between the need for specific, tailored messaging relevant to EMCs and the need to ensure messaging is not blaming and discriminatory because it is specific to certain communities and cultural practices. This leaves a problem for official information sources. The most obvious solution to this problem is to ensure proper co-production of such risk messaging with the communities to which it is targeted.

Young people in particular perceived the tone of the small amount of PA and healthy eating messaging they found during the pandemic to be “conflicting and antagonistic.” For example, the re-opening of fast-food restaurants whilst gyms remained shut was seen to be at odds with the instruction to eat healthily and keep active; or the perceived use of a “*don’t do it except you really have to*” tone in PA messages. Young people from Quebec in a study of their perceptions of public health communication during the pandemic [78] also recognised the non-verbal aspects of messaging, including the messaging tone and how they sometimes were stigmatising. The importance of tone and other non-verbal messages when developing or conveying public health messages is clear; messages from the government that clash may lead to confusion, dissatisfaction and all further messaging being ignored.

Learning from these discussions, we summarise six recommendations (six Ts) for improving the impact of public health messaging to EMCs (Table 5).

**Table 5:** Recommendations for improving the impact of public health messaging to EMCs.

Topic	Recommendation
**Together**	Togetherness underpins all other recommendations. To facilitate greater acceptance of public health messaging, co-production with community and religious group should be adopted as a strategy by the government, building on trust that already exists within these groups.
Trust	Prioritise actions to rebuild trust within EMCs by actively working with and building relationships with community and religious groups and leaders. Engage them in communicating health messaging to their communities. These actions should be consistent and sustained efforts demonstrating commitment to trust building.
Tailoring	Tailor health promoting messages to the needs and realities of EMCs. This tailoring goes beyond just mentioning these communities or including images of them in messaging. Communities need to be actively involved in the development and dissemination of the messaging to avoid the risk of messaging perceived as stigmatising.
Timing	Work with social media platforms and community groups to provide speedy, reliable, and accurate information targeted to relevant communities. This messaging needs to be swiftly produced to avoid the risk of people being exposed to persuasive misinformation as the first thing they hear or read. It also needs to be clear and precise to prevent contradiction.
Tone	Message needs to be considered carefully. This may best be done by co-producing messages with specific ethnic minority communities.
Truth	Government and public health authorities should be true to the messaging they are promoting and act in a way that is consistent with that messaging.

### Strengths and limitations

The scoping review method used enables the inclusion of records from more publicly accessible sources, relevant to a review of public perceptions of public health messages. Social media messages and audio-visuals were excluded; as significant sources of publicly consumed information, this may represent a limitation of the review. The qualitative study addressed this shortcoming by asking participants to think about all forms of messaging and were given examples that included research articles, posters, videos, TV ads and banners at bus-stops to aid recall. The findings from the qualitative study did not reveal any substantially different categories of messages than those identified by the scoping review.

Qualitative data was collected between July 2021 and March 2022, after the first and second UK COVID-19 waves. It is possible that pandemic experiences and responses had changed between these times, resulting in an inaccurate reflection of earlier COVID-19 experiences. Also, due to the extended data collection period, the recall frame may differ from participants based on their interview date. Participants’ access to and opinions on messaging may also be subject to recall bias. Time was allowed, however, for reflection and use of probing questions to facilitate recall. This resulted in some participants mentioning other health messaging they accessed, most of which were COVID-19 vaccination and IPC messaging and not on PA or healthy eating. This suggests that recall bias may have had little impact on the findings.

As is common with qualitative studies, findings from this study cannot be widely generalised. The study sample included men and women from all adult age groups (≥16) and from all ONS (Office for National Statistics, UK) main groups of EMCs. Some population groups were, however, more represented than others; there were more people from Black ethnicities, more people living in the South of England and South Wales and all the teenagers were young men. There are likely to be biases in the data which relate to the specific experiences of these groups. This study does, however, provide a basis from which to explore how messages are being received by UK EMCs. In addition, a better understanding of participants characteristics including their socio-economic status may have resulted in a more robust analysis and understanding of the data. Data collection was carried out during the peak of the COVID-19 pandemic however, during high profile conversations about inequalities, racial discrimination, and George Floyd’s death. The study team encountered considerable distrust while trying to engage with ethnic minority communities. As a result, the decision was made not to request any information that would make people think they were being chosen on the basis of specific characteristics other than ethnicity or to ask them for information they were not willing to share. For these reasons, socio-economic data was not collected. An important strength of the study is the active CE (often called Patient and Public Involvement) embedded in the research process from start to finish. This process of genuine engagement helped to build trust with EMCs leading to more open and honest conversations, and a better research environment for both researchers and study participants.

## Conclusion

PA and healthy eating messaging specifically targeting EMCs during the COVID-19 pandemic was limited and not well received. The tone of the messaging, government actions during the pandemic, negative interpretations of messaging and previous negative experiences all contributed to EMCs dismissing and distrusting the messaging they received. The most important activities to improve public health messaging and the way EMCs receive those messages is to commit to real, long-term engagement focused on trust building and to produce relevant, specific, and credible support for PA and healthy eating for EMCs. All these have great potential for improving health and wellbeing and reducing inequalities.

## Supporting information

S1 Checklist Completed PRISMA-ScR checklist for scoping review. (PDF)

S1 Text MEDLINE search strategy for scoping review. (PDF)

S1 Table Grey literature search strategy for scoping review. (PDF)
