# Supplementary material for: Misaligned or misheard? Physical activity and healthy eating messaging to ethnic minority communities during the COVID-19 pandemic: A qualitative study and scoping review
Source: PLOS Glob Public Health. 2024 Oct 3;4(10):e0003345. doi: 10.1371/journal.pgph.0003345 (PMC11449325; doi:10.1371/journal.pgph.0003345)
Supplement: S1 Text — (PDF) [file pgph.0003345.s002.pdf]

S1 Text: MEDLINE Search strategy for scoping review

Ovid MEDLINE(R) <1996 to October Week 3 2021>

1. Minority Groups/ or Minority Health/ or Ethnic Groups/ or Asian Continental Ancestry Group/ or African Continental Ancestry Group/ or Continental Population Groups/ or Culture/
2. (ethnic* adj1 minorit*).ti,ab,kw,kf.
3. (Africa* or Asia*).ti,ab,kw,kf.
4. (Mixed adj1 ethnic*).ti,ab,kw,kf.
5. (Multiple adj1 ethnic*).ti,ab,kw,kf.
6. (Black* adj1 ethnic*).ti,ab,kw,kf.
7. (BAME* or BME* or rac* or cultur* or underserv* or disadvantag* or depriv* or inequal* or marginali* or vulnerab* or Caribbean*).ti,ab,kw,kf.
8. (migrant* or immigrant* or Refugee* or (Asylum* adj1 seek*)).ti,ab,kw,kf.
9. Coronavirus/ or Coronavirus Infections/ or COVID-19/ or Pandemics/ or Quarantine/ or Patient Isolation/
10. (coronavirus* or coronovirus* or coronavirinae* or CoV).ti,ab,kw,kf.
11. ("2019-nCoV*" or 2019nCoV* or "19-nCoV*" or 19nCoV* or nCoV2019* or "nCoV-2019*" or nCoV19* or "nCoV-19*" or "COVID-19*" or COVID19* or "COVID-2019*" or COVID2019* or "HCoV-19*" or HCoV19* or "HCoV-2019*" or HCoV2019* or "2019 novel*" or Ncov* or "n-cov" or "SARS-CoV-2*" or "SARSCoV-2*" or "SARSCoV2*" or "SARS-CoV2*" or SARSCov19* or "SARS-Cov19*" or "SARSCov-19*" or "SARS-Cov-19*" or SARSCov2019* or "SARS-Cov2019*" or "SARSCov-2019*" or "SARS-Cov-2019*" or SARS2* or "SARS-2*" or SARScoronavirus2* or "SARS-coronavirus-2*" or "SARScoronavirus 2*" or "SARS coronavirus2*" or SARScoronovirus2* or "SARS-coronovirus-2*" or "SARScoronovirus 2*" or "SARS coronovirus2*" or covid).ti,ab,kw,kf.
12. (stay* adj1 home*).ti,ab,kw,kf.
13. "severe acute respiratory syndrome*".ti,ab,kw,kf.
14. (pandemic* or lockdown).ti,ab,kw,kf.
15. Policy/ or Public Policy/ or Guideline/ or Government regulation/ or World Health Organization/
16. Health Literacy/ or Health Promotion/ or Health Policy/ or Health Behavior/ or Health Knowledge, Attitudes, Practice/ or Health Communication/ or Health Education/ or International Health Regulations/ or Patient Education as Topic/ or Consumer Health Information/
17. Mental health/ or Public health/ or Population health/ or Health/ or Public Health Practice/
18. "Diet, Food, and Nutrition"/ or Diet, Healthy/ or Diet, Reducing/ or Diet Therapy/ or Diet/ or Diet, Western/ or Diet Fads/
19. Food Preferences/ or Food/
20. Nutrients/ or Nutrition Policy/ or Nutrition Therapy/ or Nutritional Requirements/ or Nutritional Status/ or Nutritional Support/ or Malnutrition/
21. Eating/ or Feeding Behavior/
22. Weight Loss/ or Overweight/ or Obesity/ or Obesity Management/
23. (food* or eat* or diet* or meal* or nutri*).ti,ab,kw,kf.
24. (nutri* adj1 immun*).ti,ab,kw,kf.
25.Exercise/
26. Physical Fitness/
27. Sedentary Behavior/

28. ((physical* adj1 activ*) or (exercis* or sport*) or active liv* or active* lifestyle* or (work* adj1 out*)).ti,ab,kw,kf.
29. ((health* adj1 liv*) or health* lifestyle*).ti,ab,kw,kf.
30. ("physical* activity*" adj1 (advi* or recommend* or tips or briefing* or consideration* or guidance* or guideline* or messag* or statement* or myth* or report* or policy* or policies or regulati* or promot* or inform* or communicat*)).ti,ab,kw,kf.
31. (nutri* adj1 (advi* or recommend* or tips or briefing* or consideration* or guidance* or guideline* or messag* or statement* or myth* or report* or policy* or policies or regulati* or promot* or inform* or communicat*)).ti,ab,kw,kf.
32. (diet* adj1 (advi* or recommend* or tips or briefing* or consideration* or guidance* or guideline* or messag* or statement* or myth* or report* or policy* or policies or regulati* or promot* or inform* or communicat*)).ti,ab,kw,kf.
33. (food* adj1 (advi* or recommend* or tips or briefing* or consideration* or guidance* or guideline* or messag* or statement* or myth* or report* or policy* or policies or regulati* or promot* or inform* or communicat*)).ti,ab,kw,kf.
34. (exercis* adj1 (advi* or recommend* or tips or briefing* or consideration* or guidance* or guideline* or messag* or statement* or myth* or report* or policy* or policies or regulati* or promot* or inform* or communicat*)).ti,ab,kw,kf.
35. (sport* adj1 (advi* or recommend* or tips or briefing* or consideration* or guidance* or guideline* or messag* or statement* or myth* or report* or policy* or policies or regulati* or promot* or inform* or communicat*)).ti,ab,kw,kf.
36. 1 or 2 or 3 or 4 or 5 or 6 or 7 or 8
37. 9 or 10 or 11 or 12 or 13 or 14
38. 15 or 16 or 17
39. 18 or 19 or 20 or 21 or 22 or 23 or 24 or 25 or 26 or 27 or 28 or 29
40. 30 or 31 or 32 or 33 or 34 or 35
41. 38 and 39
42. 40 or 41
43. 36 and 37 and 42
44. limit 43 to yr="2019 - 2021"
