# Supplementary material for: Misaligned or misheard? Physical activity and healthy eating messaging to ethnic minority communities during the COVID-19 pandemic: A qualitative study and scoping review
Source: PLOS Glob Public Health. 2024 Oct 3;4(10):e0003345. doi: 10.1371/journal.pgph.0003345 (PMC11449325; doi:10.1371/journal.pgph.0003345)
Supplement: S1 Table — (PDF) [file pgph.0003345.s003.pdf]

S1 Table: Grey Literature search strategy for scoping review

Table 1: Grey Literature resources searched that yielded articles

**Website or Resources searched**	**Search terms**
NICE Evidence Search	(Covid19 OR pandemic OR lockdown OR isolation OR quarantine) AND (BAME OR BME OR "ethnic minority") AND (nutriti* OR diet* OR immun* OR sport* OR food OR nutrient* OR exercis* OR "physical activit*" OR obesity OR sedentary)
WHO Global literature on coronavirus disease database	(Covid19 OR pandemic OR lockdown OR isolation OR quarantine) AND (BAME OR BME OR "ethnic minority") AND (nutriti* OR diet* OR immun* OR food OR nutrient* OR exercis* OR sport* OR "physical activit*" OR obesity OR sedentary)
The Kings Fund	nutriti* OR diet* OR immun* OR sport* OR food OR nutrient* OR exercis* OR physical activ* OR obesity OR sedentary AND (BAME or BME or Ethnic OR minorit) AND (Covid19 OR pandemic OR lockdown OR quarantine)
Patient UK	(Black OR Asian OR BAME OR BME OR "Ethnic minority") AND (COVID 19) AND (diet OR food OR nutrition OR nutrient OR immun OR "physical activity" or exercis OR sport OR obesity OR sedentary)
WHO Coronavirus disease advice for the public, Diabetes UK, British lung foundation, Mental health foundation (6), Migrant information Hub (2), Partnership for Maternal, New-born and Child Health, The Health foundation, British heart foundation, Google search, British Nutrition Foundation, UNICEF tips and guidance for families	Hand searched using: healthy eating, physical activity, exercise, sedentary, nutrition, diet, food, BAME, BME, ethnic, ethnic minority and COVID.
